# A computational model to simulate spectral modulation and speech perception experiments of cochlear implant users

**DOI:** 10.3389/fninf.2023.934472

**Published:** 2023-03-09

**Authors:** Franklin Alvarez, Daniel Kipping, Waldo Nogueira

**Affiliations:** ^1^Medizinische Hochschule Hannover, Hannover, Germany; ^2^Cluster of Excellence “Hearing4All”, Hannover, Germany

**Keywords:** computational model, cochlear implant, neural health, sound coding strategies, speech-in-noise recognition, spectral modulation detection, speech understanding prediction

## Abstract

Speech understanding in cochlear implant (CI) users presents large intersubject variability that may be related to different aspects of the peripheral auditory system, such as the electrode–nerve interface and neural health conditions. This variability makes it more challenging to proof differences in performance between different CI sound coding strategies in regular clinical studies, nevertheless, computational models can be helpful to assess the speech performance of CI users in an environment where all these physiological aspects can be controlled. In this study, differences in performance between three variants of the HiRes Fidelity 120 (F120) sound coding strategy are studied with a computational model. The computational model consists of (i) a processing stage with the sound coding strategy, (ii) a three-dimensional electrode-nerve interface that accounts for auditory nerve fiber (ANF) degeneration, (iii) a population of phenomenological ANF models, and (iv) a feature extractor algorithm to obtain the internal representation (IR) of the neural activity. As the back-end, the simulation framework for auditory discrimination experiments (FADE) was chosen. Two experiments relevant to speech understanding were performed: one related to spectral modulation threshold (SMT), and the other one related to speech reception threshold (SRT). These experiments included three different neural health conditions (healthy ANFs, and moderate and severe ANF degeneration). The F120 was configured to use sequential stimulation (F120-S), and simultaneous stimulation with two (F120-P) and three (F120-T) simultaneously active channels. Simultaneous stimulation causes electric interaction that smears the spectrotemporal information transmitted to the ANFs, and it has been hypothesized to lead to even worse information transmission in poor neural health conditions. In general, worse neural health conditions led to worse predicted performance; nevertheless, the detriment was small compared to clinical data. Results in SRT experiments indicated that performance with simultaneous stimulation, especially F120-T, were more affected by neural degeneration than with sequential stimulation. Results in SMT experiments showed no significant difference in performance. Although the proposed model in its current state is able to perform SMT and SRT experiments, it is not reliable to predict real CI users' performance yet. Nevertheless, improvements related to the ANF model, feature extraction, and predictor algorithm are discussed.

## 1. Introduction

People diagnosed with severe or profound sensorineural hearing loss that keep some healthy auditory nerve fibers (ANFs) are good candidates to receive a cochlear implant (CI) and recover to some extent their sense of hearing. A CI consists of an electrode array implanted in the cochlea, and a wearable sound processor usually located behind the ear. The sound processor is responsible for converting acoustic signals into electric stimulation patterns that are delivered to the ANFs *via* the intracochlear electrodes (Wouters et al., 2015). In many auditory tasks, there is a big gap in performance between normal hearing (NH) and CI listeners (Nelson et al., 2003; Nelson and Jin, 2004). Electric stimulation has its limitations to convey the necessary information for the proper coding of sounds in the auditory system (Moore, 2003). To reduce this gap, researchers are dedicated to find better CI sound coding strategies (Nogueira et al., 2005, 2009; Landsberger and Srinivasan, 2009; Dillon et al., 2016; Langner et al., 2020a; Gajecki and Nogueira, 2021), but the evaluation of the potential benefits of new ideas usually requires extensive testing procedures with implanted volunteers. In addition, there is high variability in the performance among CI users (Moberly et al., 2016), which makes it more difficult to generalize from the results.

CI sound coding strategies using current steering aim at providing an increased number of stimulation places in the implanted cochlea (Landsberger and Srinivasan, 2009; Nogueira et al., 2009). The general idea is to create virtual channels by “steering” the electrical field between two adjacent electrodes, balancing their output current at different ratios. The commercial sound coding strategy HiRes with Fidelity120 (F120), from Advanced Bionics, offers up to 120 virtual channels using 16 electrodes because every electrode pair is able to steer the electrical field to eight different locations. Furthermore, power savings can be achieved by stimulating various virtual channels simultaneously. Simultaneous stimulation allows to increase the pulse duration and consequently decrease the maximum current needed (Langner et al., 2017). The drawback is that simultaneous stimulation produces electric interaction that causes spectral smearing across channels, which also causes temporal smearing since temporal modulations may be reduced (Nogueira et al., 2021). The balance between power savings and CI users' performance was investigated by Langner et al. (2017) using three variations of the F120. Sequential stimulation (F120-S), where one virtual channel was active at a time, was compared to paired (F120-P) and triplet (F120-T) stimulation, where two and three virtual channels were active at the same time, respectively. They found out that the channel interaction that occurs with the simultaneous stimulation in F120-P and F120-T has a negative impact on performance, with F120-T obtaining the worst score. Nevertheless, high inter-subject variability was found in speech intelligibility and spectral modulation detection threshold.

It has been shown that peripheral aspects such as neural health condition (Nadol, 1997), insertion depth and position of the electrode array (Dorman et al., 1997), along with more central aspects such as neural plasticity (Han et al., 2019) may account for an important part of the inter-subject variability observed in CI users. However, it is not possible to estimate the degree of ANF degeneration without invasive methods unless the individual is already implanted with a CI (Prado-Gutierrez et al., 2006; Ramekers et al., 2014; Imsiecke et al., 2021; Langner et al., 2021). Langner et al. (2021) investigated the hypothesis that individuals with good neural health and electrode positioning will show a lower difference in performance when using simultaneous stimulation strategies (F120-P and F120-T) compared to sequential stimulation (F120-S). Healthy conditions lead to lower focused thresholds and less channel interaction between virtual channels; therefore, healthy neural conditions could lead to less detriment in performance when comparing sequential and simultaneous stimulation. The performance was evaluated using the Hochmair–Schulz–Moser (HSM) sentence test (Hochmair-Desoyer et al., 1997) at a signal-to-noise ratio (SNR) where participants roughly understood 50% of the words with F120-S. The results showed no correlation between any measure intended to estimate the neural health and difference in performance, arguably, because of the small number of individuals measured. On the contrary, computational models that simulate the electrode–nerve interface in CI users can assess the relation between neural health and performance. These computational models can isolate the parameter of study to remove the inter-subject variability, i.e., nerve count, nerve degeneration, electrode position, or insertion depth.

Computational models of the electrode–nerve interface for CIs have been proposed at different levels of complexity. Fredelake and Hohmann (2012) presented a one-dimensional interface model where the ANFs are equally distributed along a cochlear axis with the electrode array positioned in the center of this ANF population with equidistant electrodes. The spatial spread of stimulation was calculated depending on the distance between electrodes and ANFs with an exponential decay function. Neural health conditions with this model were assessed by changing the ANF density while maintaining the total neural activity constant. Lower ANF density requires higher current levels; therefore, the excitation from a single electrode reaches further ANFs in the cochlear axis causing channel interaction and spectral smearing. However, this electrode–nerve interface is very limited when representing physical aspects that occur in real implantation. From clinical imaging data, a patient-specific three-dimensional model of the implanted cochlea can be constructed (Rattay et al., 2001; Stadler and Leijon, 2009; Kalkman et al., 2014; Malherbe et al., 2016; Nogueira et al., 2016; Heshmat et al., 2020, 2021; Croner et al., 2022). These models fit a population of ANFs (type 1 spiral ganglion neurons) that extend from the organ of Corti to the central axons. Also, it is possible to control the positioning of the electrode array inside the scala tympani. The voltage spread produced by the electric stimulation from the electrode array can be calculated using a homogeneous model of the extracellular medium (Rattay et al., 2001; Litvak et al., 2007a; Nogueira et al., 2016), or using a finite element method (FEM) to account for the different electrical properties of all structures between the stimulating electrode and the ANF population (Nogueira et al., 2016). Such an electrode–nerve interface can be coupled with an ANF model capable of simulating action potentials (also called spikes) from the electrical stimulation (Ashida and Nogueira, 2018).

Regarding the ANF models, there are two different approaches. The “physiological” approach aims to simulate processes on a microscopic level. An example is the multi-compartment Hodgkin–Huxley model (Rattay, 1999; Rattay et al., 2001; Smit et al., 2008) that offers a very precise electrical behavior of the ANF segments when transmitting the action potentials throughout the peripheral axon, the soma, and the central axon. The drawback of this approach is the high demand for computational resources. The “phenomenological” approach tries to reproduce the effective outcome without detailed simulation of the involved intermediate processes. The spike generation algorithm does not consider how the spike travels through the nervous system, hence there is no geometric information involved. Some models depend completely on a probabilistic function (Bruce et al., 1999), while others are based on a leaky integrate-and-fire electrical circuit where the membrane voltage is calculated for every time step, and when it reaches a threshold, the ANF produces an action potential (Hamacher, 2004; Joshi et al., 2017). The membrane voltage depends on many other parameters like feedback currents, refractory periods, and membrane noise to introduce stochasticity. These parameters can be adjusted to fit data measured in humans or animal models to account for physiological aspects. The output of an ANF model is the “spike train”, which consists of a binary array indicating the time frames where an action potential (spike) is produced. With a population of ANFs, it is possible to integrate the spike trains, in time and cochlear place, to obtain features that are representations of sound at higher levels in the auditory system. Integration allows to reduce the amount of data while preserving the information that reaches, for example, a speech recognition algorithm (Fredelake and Hohmann, 2012; Jürgens et al., 2018).

The simulation framework for auditory discrimination experiments (FADE) (Schädler et al., 2016) is a computational tool capable of performing speech recognition tasks and psychoacoustic experiments simulating human performance. Originally, FADE was used to simulate the performance of NH and hearing aided people (Kollmeier et al., 2016; Schädler et al., 2018). Then, a CI sound coding strategy and a CI auditory model were incorporated into FADE to perform simulations of speech reception thresholds (SRTs) using data from different CI users (Jürgens et al., 2018). The SRT is defined as the signal-to-noise ratio (SNR) where 50% of the words in a sentence are correctly identified (Wagener et al., 1999) and it is a direct indicator of the CI user performance in speech understanding. However, Jürgens et al. (2018) used the same peripheral auditory model as Fredelake and Hohmann (2012), which is a simplified one-dimensional representation of the electrode–nerve interface. The incorporation of a more complex peripheral auditory model with a three-dimensional representation of the electrode–nerve interface should turn FADE into a powerful framework to assess studies related to neural health conditions in CI users. It can also be useful to assess the benefits of novel sound coding strategies. Objective instrumental measures commonly used for this purpose rely on vocoders to simulate the degraded sound delivered by the CI (Chen and Loizou, 2011; Santos et al., 2013; El Boghdady et al., 2016), not accounting for physiological aspects of the implantation.

Performance with a CI may be also predicted using simpler behavioral measurements than the SRT. The spectral modulation threshold (SMT) is defined as the ripple depth in dBs at which 79.4% of spectral rippled noise is differentiated from flat noise. SMT has been used alongside speech recognition experiments because it is a good indicator of how well the spectral cues in speech signals were perceived (Litvak et al., 2007b; Langner et al., 2017). It was used by Langner et al. (2017) as an indicator of how these spectral cues are affected by the channel interaction occurring in simultaneous stimulation (F120-P and F120-T), compared to sequential stimulation (F120-S). Their results showed similar performance between F120-S and F120-P but a clear lowering of performance with F120-T.

In this study, a computational model that simulates the performance of real CI users in SRT and SMT experiments is presented. The goal is to show the effects of parallel stimulation and neural degeneration in CI outcome performance. This model was tested with the three sound coding strategies used by Langner et al. (2021) (F120-S, F120-P and F120-T), and the hypothesis that channel interaction affects individuals with poorer neural health conditions to a larger extent is assessed. In the next section, the different parts composing this computational model and how the SRT and SMT experiments were implemented with FADE are described. A further section presents the results obtained, and the last section contains the discussion and conclusions of this study.

## 2. Materials and methods

### 2.1. The computational model

The proposed computational model consists of (i) a “front-end” containing the CI sound coding strategy, the peripheral auditory model with a three-dimensional electrode–nerve interface, and a feature extraction algorithm; (ii) a “back-end” with a hidden Markov model (HMM) already incorporated in the framework FADE.

#### 2.1.1. Front-end

##### 2.1.1.1. Cochlear implant sound coding strategy

**Figure 1:**
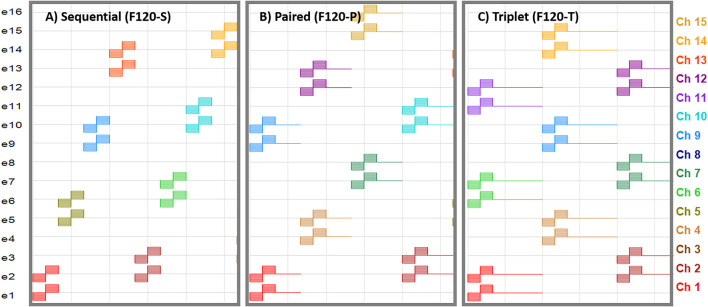
Pulse tables for the sequential, paired, and triplet stimulation versions of the HiRes Fidelity120 sound coding strategy. Horizontal axis is the temporal axis. Electrodes are enumerated from e1 (most apical) to e16 (most basal). The virtual channels composed of two simultaneously stimulated electrodes are enumerated from channel 1 (Ch 1; stimulation with e1 and e2) to channel 15 (Ch 15; stimulation with e15 and e16). **(A)** Sequential stimulation, where the virtual channels are activated one after another. **(B)** Paired stimulation, where two virtual channels are activated at the same time followed by a zero-phase gap with an equivalent duration of one biphasic pulse. **(C)** Triplet stimulation, where three virtual channels are activated at the same time followed by a zero-phase gap with an equivalent duration of two biphasic pulses.

The software BEPS+ from Advanced Bionics was used to create the pulse tables for the F120-S, F120-P, and F120-T sound coding strategies. The pulse tables are defined as the sequence of electrical pulses to create one cycle of stimulation. Figure 1 shows partial pulse tables corresponding to these strategies. A pulse consists of a cathodic-leading biphasic pulse. The electrodes are enumerated from the most apical to the most basal and each virtual channel composed of two simultaneously stimulated electrodes are depicted with a color code. The pulse phase duration was set to 18 μs and the pulse rate across virtual channels was kept constant at 1,852 pps by adding a gap between subsequent pairs, or triplets, of stimulating virtual channels for F120-P and F120-T, respectively.

The HiRes implantable cochlear stimulator (ICS) from Advanced Bionics was used to transform audio signals into electrodograms. The audio signal was calibrated to −49 dB full scale [dB_FS_], corresponding with an audio signal at 65 dB sound pressure level [dB_SPL_] captured by the microphone of the CI device. At this value, the signal level was close to the knee point of the adaptive gain control of the CI sound processor.

Biphasic pulses of the electrodograms were resampled to 1 MHz to guarantee equal anodic and cathodic phases. This sample rate was also needed in the implementation of the peripheral auditory model.

##### 2.1.1.2. Peripheral auditory model

**Figure 2:**
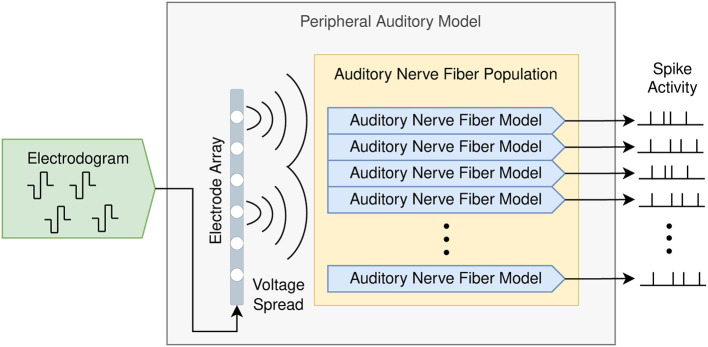
Peripheral auditory model for cochlear implants consisting of a population of auditory nerve fibers and an electrode array. The input is the electrodogram generated by the cochlear implant sound coding strategy, and the output is the spike activity produced by the auditory nerve fiber population.

Figure 2 shows the composition of the proposed peripheral auditory model. The electrodograms obtained from the sound coding strategy were transformed to obtain the voltage spread based on a three-dimensional electrode–nerve interface model embedded in a homogeneous medium. The amount of stimulation at every ANF was obtained from this voltage spread and the times when action potentials are elicited in every ANF (spike trains) were simulated with an active nerve fiber model. The spike activity is defined as the collection of spike trains produced in an ANF population.

**Figure 3:**
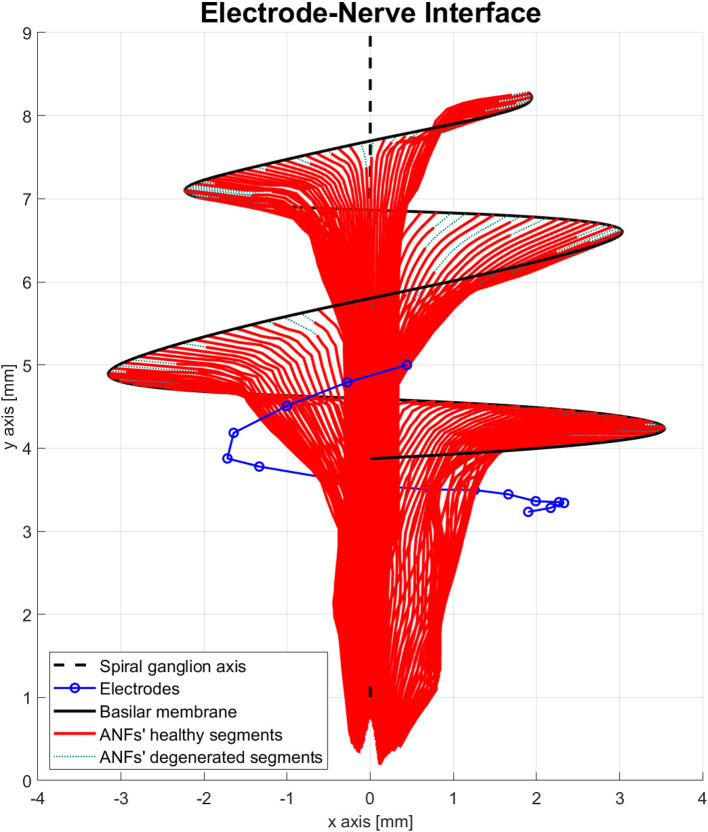
Electrode–nerve interface. It is a three-dimensional representation of the auditory nerve fibers (ANFs) (red) arranged in a spiral shape along the basilar membrane (continuous black line) and centered in the spiral ganglion axis (dashed black line). The electrodes (blue) are inserted almost one complete turn into the scala tympani. Some ANFs present a mild degeneration (dotted green).

The electrode–nerve interface used in the proposed model was based on the cochlea model presented in Nogueira et al. (2016). Cochlear geometry, electrode location, and position of the ANF population were taken from a generic version of their model. The number of ANFs was increased from 7,000 to 9,001 and distributed along 900° of insertion angle from base to apex (two turns and a half) with a separation of 0.1°. The ANFs were indexed in order from the base of the cochlea (high frequencies) to the apex of the cochlea (low frequencies). Another adjustment was done to the electrode array. Nogueira et al. (2016) modeled an electrode array of 22 electrodes; therefore, the electrodes 21, 19, 17, 15, 13, and 11 were removed to obtain the 16 electrodes present in advanced bionics CIs. The resulting electrode–nerve interface is shown in Figure 3.

The morphology of the ANFs was modeled after the myelinated fibers presented in Ashida and Nogueira (2018), which is a simplified representation consisting of segments with a constant internodal length (*L*_*i*_) equal to 200 μm that extends from the location of the peripheral terminal toward the cochlear nerve. In this morphological model, there is no differentiation between the peripheral axon, central axon, or the soma. As in Ashida and Nogueira (2018), the electric stimulation in the myelinated model was calculated at the nodes that join together two adjacent segments. The voltage produced by the stimulation current *I*_*n*_ coming out of the electrode *n* was calculated for every node *a* of every fiber *f* as shown in Equation (1).


(1) $$\begin{array}{l} U_{nf_{a}}=\frac{\rho_{\text{ext}}I_{n}}{4\pi d_{nf_{a}}}. \end{array}$$

The extracellular resistivity of the homogeneous medium (ρ_ext_) was set to 3.0 Ωm as in Ashida and Nogueira (2018). The variable *d*_*n*_*f*__*a*__ is the distance between the electrode *n* and the node *a*. This approach results in a voltage spread inversely proportional to the distance *d*_*n*_*f*__*a*__ (Litvak et al., 2007a; Nogueira et al., 2016).

The activation function has been proposed by Rattay (1999) to approximate the amount of functional electrical stimulation over an ANF. In this model, it was calculated as shown in Equation (2). The activation function in a node *a* depends on its external voltage (*U*_*n*_*f*__*a*__), and the external voltage on its adjacent nodes (a-1 toward the periphery and a+1 toward the central neural system). The axon internal resistance (*R*_*i*_) was obtained as “$R_{i}=4L_{i}r/\pi D^{2}$”. The axon diameter (*D*) was set to 2.0 μm, and the axial resistivity (*r*) to 1.0 Ωm, as mentioned by Ashida and Nogueira (2018). Notice that the activation function in this study has units in Amperes [A] instead of volts per second [V/s] as originally defined by Rattay (1999). This is because the membrane capacitance is not included in Equation (2); however, this membrane capacitance is taken into account in a further stage of the model.


(2) $$\begin{array}{l} A_{nf_{a}}=\frac{U_{nf_{a-1}}-U_{nf_{a}}}{R_{i}}+\frac{U_{nf_{a+1}}-U_{nf_{a}}}{R_{i}}. \end{array}$$

To simulate the spikes generated by each ANF, the neuron model of Joshi et al. (2017) was implemented. This is a “phenomenological” model that represents the peripheral and central axons as two independent adaptative integrate-and-fire circuits that are coupled together by a logical “OR” gate (see Figure 1 in Joshi et al., 2017). This phenomenological model does not convey any geometric information such as the distance between the stimulating electrode and the ANF. Therefore, the induced current *I* (called stimulation current in Joshi et al., 2017) was adjusted according to the activation function obtained from the electrode–nerve interface model as shown in Equation (3).


(3) $$\begin{array}{l} I=M_{C}\overset{N}{\underset{n=1}{\sum }}A_{nf_{a_{\text{max}}}}. \end{array}$$

Notice that the activation function in Equation (2) has a value for every node in an ANF. To simplify the implementation, only the node (*a*_*max*_) with the maximum absolute value of the activation function was taken into account to compute the induced current *I*. This is based on the fact that this is the node with the highest probability to produce a spike. In addition, a modeling factor (*M*_*C*_) that allowed to calibrate the peripheral auditory model was added. It was adjusted to reproduce approximately the same spike count reported by Joshi et al. (2017) given different stimulation current levels.

The model of Joshi et al. (2017) assumes that the peripheral and central circuits share the same induced current (*I*), but they respond differently to the positive (anodic; *I*^+^) and negative (cathodic; *I*^−^) phases of the biphasic pulses. Therefore, in this study, the peripheral axon circuit is referred to as cathodic-excitatory while the central axon circuit as anodic-excitatory. The circuit specific induced current (*I*_*Stim*_) was obtained with Equation (4), where the inhibitory compression (β) was set to 0.75.


(4) $$\begin{array}{l} I_{\text{Stim}}=\{\begin{array}{cc} -\left(I^{-}+\beta I^{+}\right) & \text{Cathodic-excitatory circuit.}\\ I^{+}+\beta I^{-} & \text{Anodic-excitatory circuit.} \end{array} \end{array}$$

The membrane voltage (*V*) for both circuits is calculated with Equation (5), where the membrane capacitance (*C*) takes different values for the cathodic-excitatory (856.96 nF) and the anodic-excitatory (1772.4 nF) circuit, *h*(*V*), is a passive filter dependent on membrane voltage, *I*_*Sub*_ and *I*_*Supra*_ are internal subthreshold and suprathreshold adaptation currents, and *I*_*Noise*_ is a noise current source with a Gaussian spectral shape that introduces stochastic behavior into the spike trains. The passive filtering, the evolution of the adaption currents and the noise have their own function and can be found in the publication of Joshi et al. (2017). Whenever the membrane voltage of the cathodic-excitatory or the anodic-excitatory circuit reached a threshold, a spike was generated and the ANF entered in an absolute refractory period (ARP) of 500 μs. During the ARP, neither the cathodic- nor anodic-excitatory circuit could produce a spike.


(5) $$\begin{array}{l} C\frac{dV}{dt}=h\left(V\right)-I_{Sub}-I_{Supra}+I_{Noise}+I_{Stim}. \end{array}$$

**Figure 4:**
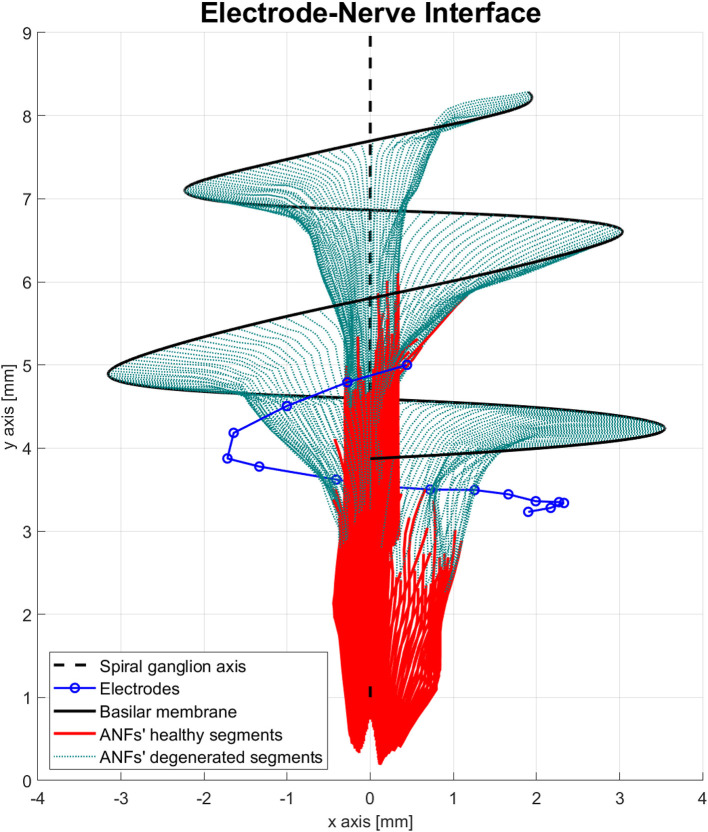
Electrode–nerve interface geometrical model with auditory fibers degenerated (dotted green). Mean degeneration (α_*f*_) equal to 20 segments.

Another important feature of the proposed peripheral auditory model is the representation of different neural health conditions. A degeneration index (α_*f*_) was assigned to every ANF, which was a natural number from 0 to 20, indicating how many segments were removed from its modeled morphology. The segments were always removed from the most peripheral part resembling the dendritic degeneration that occurs when the inner hair cells in the basilar membrane are damaged (Spoendlin and Schrott, 1988; Nadol, 1997). The nerve degeneration was limited to 20 segments because, beyond this point, the amount of stimulation current required to elicit a spike was excessive compared to real CI users. Figure 4 shows how nerve degeneration was implemented in the proposed electrode–nerve interface.

It is worth mentioning that removing segments may result in situations where the degenerated part surpasses the physical location of the soma, which in real spiral ganglion neurons would be somewhere between the seventh and the twelfth segment. Nevertheless, degeneration of the peripheral axon could also be modeled as the loss of myelin sheets, or by reducing its diameter (Heshmat et al., 2020, 2021; Croner et al., 2022). The effects of this type of degeneration would be that nodes in the central axon will be the ones that produce a spike. Therefore, removing segments in our model was used to investigate excitation at most central locations, rather than to represent the real physical degeneration.

Because it is unknown how the current flows in the most peripheral nodes after degeneration, it was decided to discard them from the activation function calculation. In this regard, Rattay (1999) proposed to remove the first term in Equation (2); nevertheless, in degenerated ANFs, following this proposal could result in a rise of the activation function despite the worst neural health condition and this effect was undesired in our model.

##### 2.1.1.3. Internal representation as features

The feature extraction algorithm was based on the internal representation (IR) presented by Fredelake and Hohmann (2012), which accounts for more central processes in the auditory pathway. The IR consists of a spatial and a temporal integration of the spike activity produced by the ANF population. The first step was to downsample the spike activity to a sample rate of 10 kHz.

To perform the spatial integration, the ANFs were grouped resembling the auditory filters described in Moore (2003). It is mentioned that a maximum of 39 independent auditory filters can be formed at the same time to code sound in NH people, but it is also mentioned that the effective number of channels for CI users could be reduced depending on the number of electrodes implanted. Therefore, in the computational model, the number of auditory filters used was limited between 16 (number of electrodes) and 39. Lengths of the auditory filters ranged between 1.1 and 2.6 mm in the 42 mm basilar membrane of the modeled cochlea. To obtain the number and distribution of these auditory filters along the basilar membrane, adjacent ANFs were grouped by their most likely stimulating electrode (highest absolute value of the activation function) to form auditory filters. If an auditory filter was below the minimum size (1.1 mm), its fibers were merged with the adjacent auditory filter toward the apex. In case an auditory filter size exceeded the maximum value (2.6 mm), its most basal ANFs were used to form a new auditory filter of maximum size while the remaining ANFs were used to form a different auditory filter. Once the auditory filters were formed, the spike trains of their corresponding ANFs were added together to obtain the spike group activity (*S*_*g*_), where *g* was the auditory filter index.

The next step was to integrate this spike group activity across time. For each group, the signal was low pass filtered as shown in Equation (6), where *F*_*g*_(*k*) is the filtered spike group activity, *k* is the time frame index, *f*_*s*_ is the sample frequency equal to 10.0 kHz, τ_*LP*_ is the time constant of the filter set to 1 ms, and the operator “*” denotes a convolution.


(6) $$F_{g}(k)=S_{g}(k)*\exp(-{\left(\frac{k}{\sqrt{2}f_{s}\tau_{LP}}\right)}^{2}).$$

From this point, a forward masking effect is implemented. A masker signal *Z*_*g*_ was derived from the filtered spike group activity using a recursive low pass filter (see Section 2.3 in Fredelake and Hohmann, 2012). This masker signal increases exponentially with onsets in the spike group activity and decreases exponentially with the offsets. The IR is finally the maximum value between the masker signal and the filtered spike group activity. The whole forward masking effect is taken from Fredelake and Hohmann (2012) and it is not detailed in this study. A visual representation is found in Figure 4 of Fredelake and Hohmann (2012).

To meet the requirements of the back-end, the IR was further downsampled to a sample rate of 100 Hz using a moving average low pass filter to diminish the effects of aliasing.

#### 2.1.2. Back-end

The computational model used the framework FADE as the back-end. As a predictor algorithm, FADE uses an HMM that represents the target stimulus with an eight-state Markov chain and a one-component Gaussian mixture model (GMM) to learn, and subsequently predict, the features (each auditory filter in the IR) (Schädler et al., 2016). FADE counts with different “ready-to-use” experiment templates, processing algorithms, and feature extraction algorithms that are intended to predict NH and hearing aid performances (Kollmeier et al., 2016; Schädler et al., 2016, 2018). Hence, two new experiment templates (described in detail in Section 2.4) were developed to perform SRT and SMT experiments. The tasks handled by these new templates are as follows:

1. The generation of the stimulus audio files composing the training and testing corpus.
2. The generation of the electrodograms from these audio files using the respective CI sound coding strategy as the processing algorithm.
3. The generation of the stimuli' IRs using the proposed peripheral auditory model as the feature extraction algorithm.
4. The training of the HMM with the IRs obtained from the training corpus.
5. The predictions over the IRs of the testing corpus with the trained HMM.
6. The evaluation of the performance of the HMM.

In the evaluation stage, FADE generated a file with the score obtained at different training conditions (dB_SNR_ in SRT experiments and dB_contrast_ in SMT experiments). Scores were represented as data points in a scatter plot and a non-linear regression to a psychometric function was performed. This psychometric function is defined in Equation (7), where *p*_*chance*_ is the lower horizontal asymptote of the function representing the probability of getting a correct answer with random predictions, *p*_*max*_ is the upper asymptote of the function representing the predicted performance in ideal conditions, *p*_*range*_ is the difference between the upper and lower asymptotes, *s* is the slope, or growth rate, at the inflection point of the psychometric function, and *x*_*o*_ is the offset of the inflection point in the x-axis (dB_SNR_ or dB_contrast_). The regression was performed with the MATLAB function “*fitnlm*”. The coefficient of determination *R*^2^ was obtained in every experiment, which is a reference of how well the scattered data was represented by the regressed psychometric function.


(7) $$\begin{array}{l} Ps\left(x\right)=p_{chance}+\frac{p_{range}}{1+e^{-s\left(x-x_{o}\right)}}. \end{array}$$

### 2.2. Fitting and calibration

The fitting procedure in CI users consists of the adjustment of the stimulation levels of each electrode in the array, or virtual channels in the case of current steering strategies such as F120. Each electrode, or virtual channel, stimulates at levels between threshold (T) and most comfortable loudness (MCL) that are unique for every CI user. By default, the advanced bionics device sets T to 10% of the MCL level resulting in a 20 dB dynamic range. Stimulation levels with the advanced bionics device are given in clinical units (CU), which are integer values from 1 to 471. The equivalent output current (*I*_*n*_) [μA] was obtained with Equation (8), where *T*_*p*_ is the pulse duration in μs (18 μs in this study), *I*_*max*_ is the maximum output current equal to 2,040 μA, and *T*_*max*_ is the maximum pulse duration equal to 229 μs (Advanced Bionics, 2020).


(8) $$\begin{array}{l} I_{n}=\frac{CU}{6000}\frac{I_{max}T_{max}}{T_{p}}. \end{array}$$

The process of fitting requires a closed feedback loop, where the CI user indicates the loudness perceived to an audiologist. This loop is virtually closed in the computational model by measuring the spike activity produced by electric pulse trains at different levels of stimulation (from 1 to 471 CU in steps of 30 CU) based on the assumption that the loudness perception is closely related to the neural activity produced by the ANFs (McKay and McDermott, 1998; McKay et al., 2001).

The fitting stimulus used was a cathodic-leading biphasic pulse train with a pulse duration of 18 μs and a periodicity of 540 μs (resulting approximately in 1,852 pps) was consistent with the experimental parameters. The pulse train had a duration of 200 ms with 10 ms of leading and preceding silence. Because the periodicity of this fitting stimulus is just above the ARP, it was expected that ANFs close to the electrodes “fired” with every biphasic pulse of the pulse train.

For each virtual channel, a group of 858 ANFs with the highest absolute activation function was selected. This number corresponds to the number of fibers found in approximately 4 mm section of the modeled basilar membrane, although the selected ANFs were not constrained to be adjacent to each other. The MCL was defined as the CU value where each biphasic pulse elicited a spike in the selected ANF group with a probability of 75%. A similar assumption was used by Kalkman et al. (2014). T level was set to the 10% of the MCL level.

The calibration of the peripheral auditory model refers to the adjustment of the modeling factor (*M*_*C*_) shown in Equation (3). This process was closely related to the fitting procedure described earlier. It was selected a *M*_*C*_ equal to 89.525 × 10^6^, which guaranteed that MCL levels did not exceed the maximum of 250 cu in any neural health condition used in the experiments. Stimulation above this limit would produce undesired out of compliance stimulation.

### 2.3. Neural health conditions

In preliminary experiments (not shown in this study) it was found that 9001 fibers introduced a considerable amount of redundant information, and also, in the majority of the ANFs the node with the highest activation function for any electrode was beyond the fifth node. Therefore, it was decided to use different α_*f*_ for every ANF, but defining a mean degeneration value with a standard deviation of three nodes. This diminished the redundant information and was a more realistic representation of how the degeneration gradually occurs (Nadol, 1997).

**Figure 5:**
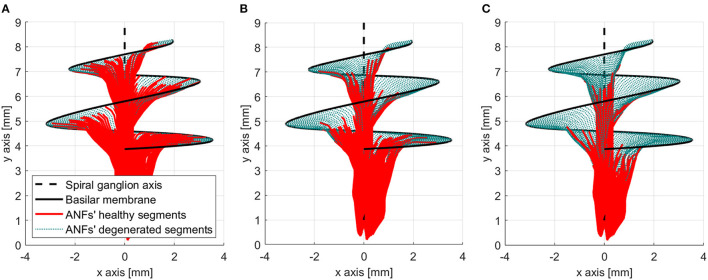
Three neural health condition representations. **(A)** Healthy conditions (mean degeneration of 5 nodes). **(B)** Moderate auditory nerve fiber (ANF) degeneration (mean degeneration of 10 nodes). **(C)** Severe ANF degeneration (mean degeneration of 15 nodes).

The peripheral models with a mean ANF degeneration of 5, 10, and 15 nodes were considered to have a “healthy” neural health condition, a “moderate” loss, and a “severe” loss, respectively. Those three cases were assessed in this study and are shown in Figure 5.

### 2.4. Experiments

A total of nine SMT experiments and nine SRT experiments were performed using the three variants of the F120 sound coding strategies (F120-S, F120-P, and F120-T) and three aforementioned neural health conditions. Each neural health condition in the peripheral auditory model can be considered as an “individual” in experiments with real CI users.

#### 2.4.1. Spectral modulation threshold experiments

The spectral modulation threshold (SMT) experiment was defined by Litvak et al. (2007b). It consists of measuring the smallest detectable spectral contrast in spectral rippled noise. The spectral shape of spectral rippled noise is sinusoidal and it is generated using Equation (9), where |*F*(*f*_*r*_)| is the magnitude at the frequency bin *f*_*r*_, *C*_*t*_ is the spectral contrast in dB, *f*_*RPO*_ is the ripple-per-octave, and θ_0_ is the ripple phase in the spectrum. Notice that the signal is hard-filtered at values below 350 Hz and above 5,600 Hz.


(9) $$|F(f_{r})|=\{\begin{array}{ll} 10^{\frac{C_{t}}{2}\sin(2\pi (\log_{2}(f_{r}/350))f_{RPO+}\theta_{0})/20} & 350<f_{r}<5600\\ 0 & otherwise \end{array}$$

With human participants, the SMT is obtained with a three intervals two alternative forced choice procedure consisting of two reference intervals with no ripple (*C*_*t*_ equals to 0), and one target interval with a defined *C*_*t*_. The first interval is always a reference noise, hence the participant has to indicate if the target interval is presented in the second or third position. This adaptive procedure is detailed by Litvak et al. (2007b), and has an equilibrium point of 79.4% correct answers. Because FADE uses a “training and testing” approach, the adaptive procedure could not be implemented; however, the equilibrium point is kept as the detection threshold.

The corpus was generated using Equation (9) with MATLAB. The ripple phase (θ_0_) was randomly selected for every stimulus signal. A ripple per octave (*f*_*RPO*_) equal to 0.5 was selected as in the experiments from Litvak et al. (2007b) and Langner et al. (2017).

The training corpus consisted of 1,000 samples of the spectral ripple noise with random integer values between 2 and 20 dB for contrast depth, and 1,000 samples of reference noise (*C*_*t*_ equals to 0). The testing corpus consisted of 10 sets, each one with 50 samples of reference noise and 50 samples of spectral ripple noise at a target *C*_*t*_ of 2, 3, 4, 5, 7, 9, 11, 14, 17, and 20 dB, respectively. In total 1,000 samples were predicted at 10 different contrast levels.

The sampling frequency of every sample was 17.4 kHz and the stimulus duration was limited to 0.4 s. In all cases, loudness roving was implemented keeping a mean value of −49 dB_FS_, a roving peak of 5 dB_FS_, and a roving resolution of 0.5 dB_FS_.

The spectral modulation detection performance was described by the psychometric function in Equation (7), where *p*_*chance*_ was set to 50% because it was a binary decision. The SMT was the *x* value, where *Ps*(*x*) was equal to 79.4%.

#### 2.4.2. Speech reception threshold experiments

The SRT experiments were performed using the Oldenburg sentence test (OLSA). OLSA consists of a matrix sentence test of 50 words that belong to five different categories of 10 words each: name, verb, number, adjective, and noun. The sentences were constructed with one word from each category, following the previously mentioned order, giving a total of 10^5^ possible combinations (Wagener et al., 1999). In a closed test procedure, the participants have previous knowledge of the words that may appear. Several sentences, mixed with noise at a specific SNR, are presented to the subject and the subject is asked to repeat them. A score based on the percentage of correctly recognized words is obtained. This is repeated at different SNR conditions and then a psychometric function is fitted to the obtained data points. The SNR value where this psychometric function crosses the 50% mark of correctly recognized words is the SRT result.

For SRT experiments, Schädler et al. (2016) and Jürgens et al. (2018) used a subset of 120 OLSA sentences to generate the training and testing corpus, but in this study, only a subset of 100 OLSA sentences was used to reduce computational resources. In this subset, each of the 50 words in the matrix appears exactly 10 times. A random excerpt of the noise provided by OLSA was added to the sentences at the required level to obtain the different SNRs, while the speech was kept at −49 dB_FS_.

FADE uses a closed training/testing approach, meaning that the same sentences used in the training are used in the prediction stage. Therefore, the training corpus was generated with all the sentences in the subset mixed with noise at seven different SNR levels, from 0 to 18 dB in steps of 3 dB, and without noise, giving a total of 80 unique instances for each word. The testing corpus was generated with all the sentences mixed at 10 different SNR values, from −9 to 18 dB in steps of 3 dB, giving a total of 5,000 words to be predicted.

Regarding the psychometric function described in Equation (7), *p*_*chance*_ was set to 10% because it was a one word out of 10 decisions. The SRT was the *x* value where *Ps*(*x*) was equal to 50%.

## 3. Results

### 3.1. Fitting

**Figure 6:**
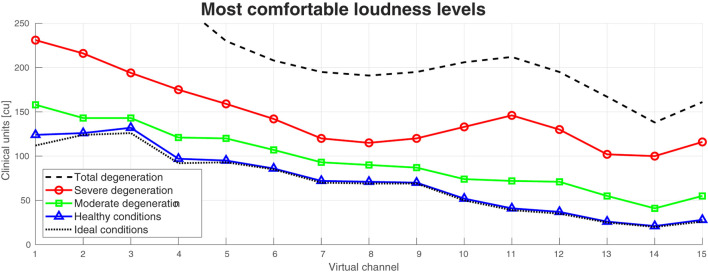
Most comfortable levels at different neural health conditions of the auditory peripheral model. Ideal conditions is equivalent to no degeneration in the auditory nerve fibers (ANFs). Healthy conditions, moderate degeneration, and severe degeneration are equivalent to a mean of 5, 10, and 15 segments degenerated in the ANFs, respectively. Total degeneration is equivalent to 20 segments degenerated in almost all the ANFs.

Figure 6 shows the MCL levels obtained for the computational model with the healthy, moderate degeneration, and severe degeneration condition. It also shows as a reference an ideal case (no degeneration in the ANFs), and the worst case (20 degenerated segments in the ANFs).

The MCL level difference across the 15 virtual channels between ideal and healthy conditions was only on average 2.87 CU. This difference increased with worse neural health conditions. Between healthy conditions and moderate degeneration, the difference was on average 23.47 CU, between moderate and severe degeneration, the difference was 51.27 CU, and between moderate and total degeneration, the difference was 80.53 CU. With total degeneration in the ANFs, the MCL levels of the four most basal electrodes (high frequencies) were above the desired 250 CU.

### 3.2. Electrical interaction

**Figure 7:**
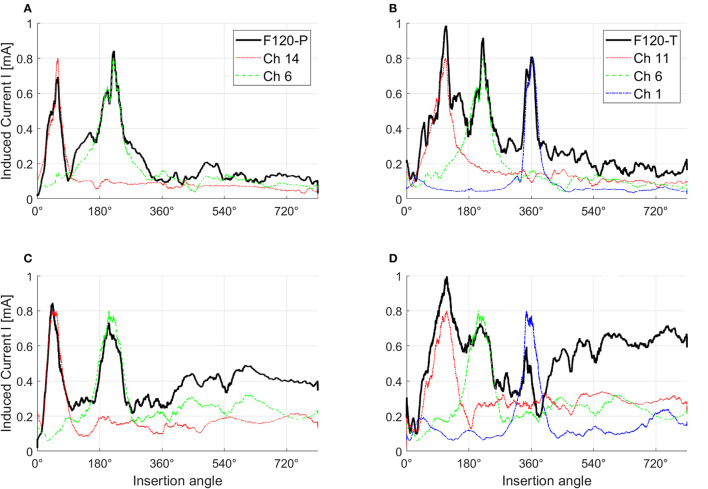
Effects of electrical interaction in simultaneous stimulation with F120-P and F120-T sound coding strategies. Panels **(A, B)** show electrical interaction in healthy condition while panels **(C, D)** show electrical interaction in severe degeneration. For this example, the stimulation pulses in every virtual channel were adjusted until they reached a peak of the induced current across the auditory nerve fibers of 0.8 mA. F120-P uses the virtual channels 6 (Ch 6) and 14 (Ch 14) simultaneously as shown in **(A, C)**. F120-T uses the virtual channels 1 (Ch 1), 6 (Ch 6), and 11 (Ch 11) as shown in **(B, D)**. The induced current with simultaneous stimulation is shown with black continuous lines. The y-axis corresponds to the magnitude of the induced current and the x-axis to the insertion angle of the electrode array from base (high frequencies) to apex (low frequencies). The figure was smoothed to provide a better visualization.

Figure 7 shows the effects of electrical interaction with simultaneous stimulation after fitting. For this figure, paired biphasic pulses were generated for the 1st, 6th, 11th, and 14th virtual channels to obtain a peak of induced current *I* across the ANF population of 0.8 mA. The black continuous lines correspond to the induced current with simultaneous stimulation, while the induced currents resulting from each channel individually (sequential stimulation) are shown with different colors. The induced current with simultaneous stimulation in healthy conditions (Figures 7A, B), and with paired stimulation in severe degeneration (Figure 7C), follows the peaks corresponding to the induced current of each individual virtual channel. This is not the case with triplet stimulation in severe degeneration (Figure 7D), where the peaks corresponding to virtual channels 6 and 14 are almost merged together while the peak corresponding to the virtual channel 1 is attenuated. Attenuation occurs when different virtual channels have opposite activation function signs; therefore, they cancel each other in Equation (3). Note that the induced current toward the apex of the cochlea (insertion angles around 540° and 720°) also increases with worse neural health conditions as a consequence of higher stimulation levels.

### 3.3. Spectral modulation threshold experiments

**Figure 8:**
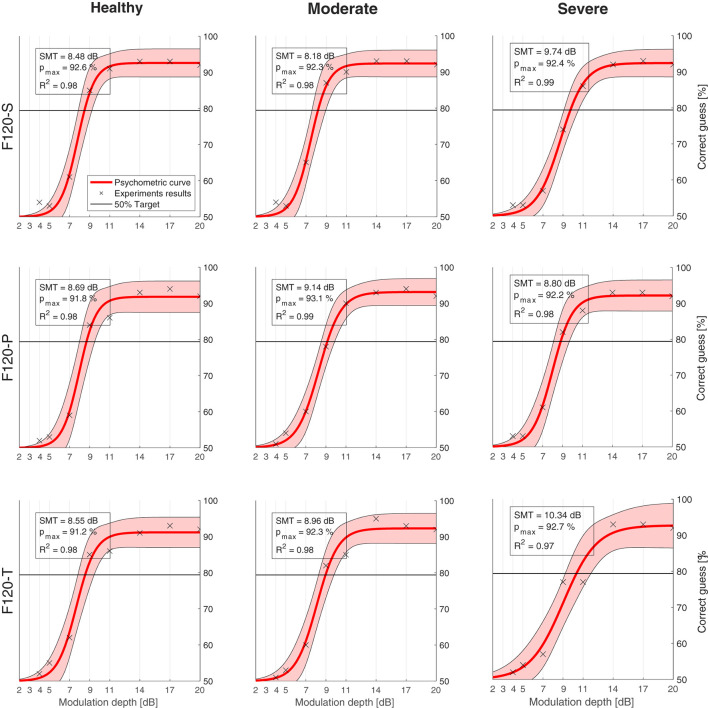
Psychometric functions (red) obtained from the computational model results (crosses). The legend in every chart indicates the spectral modulation threshold (SMT), which is the modulation depth where the psychometric function crosses the 79.4% target line (black). *p*_*max*_ is the expected performance when the modulation depth tends to infinity. *R*^2^ is the coefficient of determination between the results and the psychometric curve. The shaded area shows 95% confidence interval. Charts are arranged in columns by neural health (**left** to **right**: healthy conditions, moderate degeneration, and severe degeneration), and in rows by sound coding strategy (**top** to **bottom**: F120-S, F120-P, and F120-T).

Figure 8 shows the psychometric functions obtained from the SMT experiments. The upper left box indicates the corresponding SMT, the expected performance with an infinite modulation depth (*C*_*t*_), and the coefficient of determination (*R*^2^). In general, the psychometric regression obtained an *R*^2^ coefficient ranging from 0.97 to 0.99, which means that the psychometric function represents a good fit for the results obtained.

**Figure 9:**
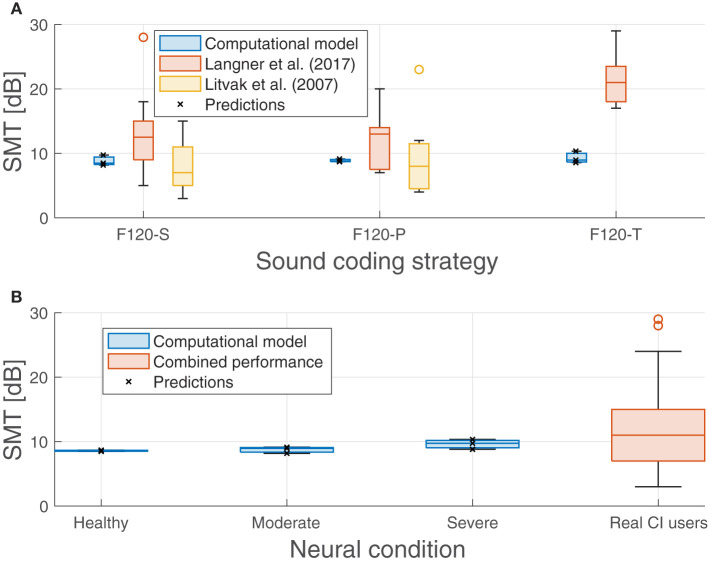
Predicted spectral modulation threshold (SMT) obtained in the experiments compared with measurements obtained with real CI users from Litvak et al. (2007a) and Langner et al. (2017). **(A)** Performance of the proposed computational model and the performance from literature grouped by sound coding strategy. **(B)** Performance of the proposed computational model grouped by neural health conditions and the combined performance from Litvak et al. (2007a) and Langner et al. (2017) as a comparison. Predicted values are shown with crosses.

Nevertheless, Figure 9 shows that there was no significant effect on the performance regarding the sound coding strategy (Figure 9A) or the neural health condition (Figure 9B). As shown in Figure 9B, a small trend toward poorer performance with worse neural health conditions (8.57 dB for healthy conditions, 8.76 dB for moderate degeneration, and 9.63 dB for severe degeneration) is not significant compared to the clinical data obtained by Litvak et al. (2007a) and Langner et al. (2017). Also, as shown in Figure 9A, the expected effect of sound coding strategy on performance is not obtained (e.g., with severe degeneration the SMT is better using F120-P than using F120-S). The *p*_*max*_ seems to not be affected either by the neural degeneration or sound coding strategy since it varies from 91.2% (F120-T at healthy conditions) and 93.1% (F120-P at moderate degeneration of the ANFs).

Data from Litvak et al. (2007b) were collected from 25 CI users (Saoji et al., 2005) and are shown in Figure 9, part A, with blue boxes. The CI users were using F120-S (14 participants) and F120-P (11 participants) sound coding strategies. The results from Langner et al. (2017), shown in the same graph with green boxes, included 14 experiments. Half of them showed the SMT comparison between F120-S and F120-P sound coding strategies, while the other half was between F120-S and F120-T. Their performance showed a large variability but the trend is to get worse with simultaneous stimulation, especially with F120-T.

The performance of the computational model ranged from 8.48 to 10.34 dB, which can be considered as a “good” to “average” performance for CI users, but it does not account for the variability. The difference between the worst and the best performance of the model was only 1.90 dB, while in real CI users, it can be more than 18 dB.

### 3.4. Speech reception threshold experiments

**Figure 10:**
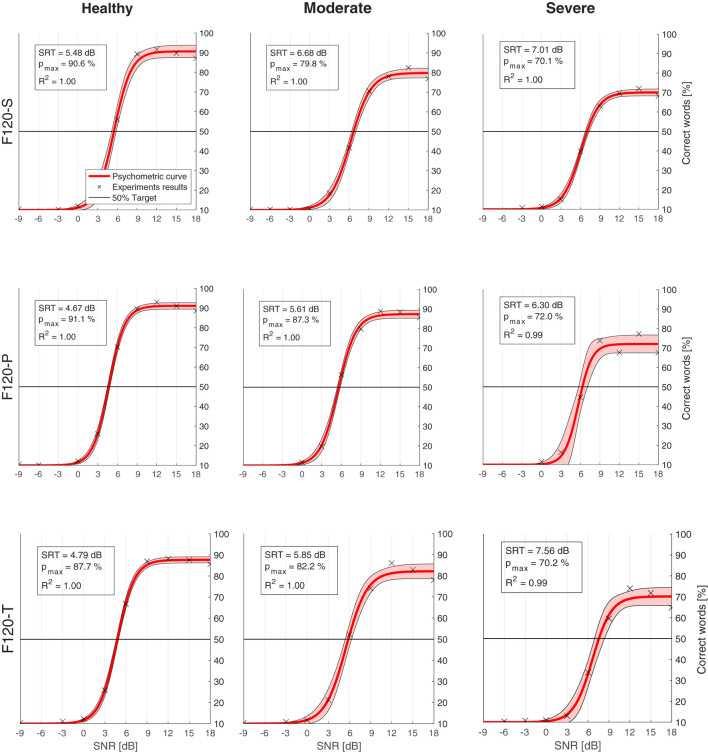
Psychometric functions (red) obtained from the computational model results (crosses). The legend in every chart indicates the speech reception threshold (SRT), which is the signal-to-noise ratio (SNR) where the psychometric function crosses the 50% target line (black). *p*_*max*_ is the expected performance in quiet. *R*^2^ is the coefficient of determination between the results and the psychometric curve. The shaded area shows 95% confidence interval. Charts are arranged in columns by neural health (**left** to **right**: healthy conditions, moderate degeneration, and severe degeneration), and in rows by sound coding strategy (**top** to **bottom**: F120-S, F120-P, and F120-T).

Figure 10 shows the psychometric functions obtained from the SRT experiments. The upper left box in every chart indicates the corresponding SRT, the expected performance “in quiet” with *p*_*max*_, and the coefficient of determination (*R*^2^) obtained with the regression. In general, the psychometric function represented quite well the data resulted from the experiments, obtaining an *R*^2^ coefficient always equal or greater than 0.99.

**Figure 11:**
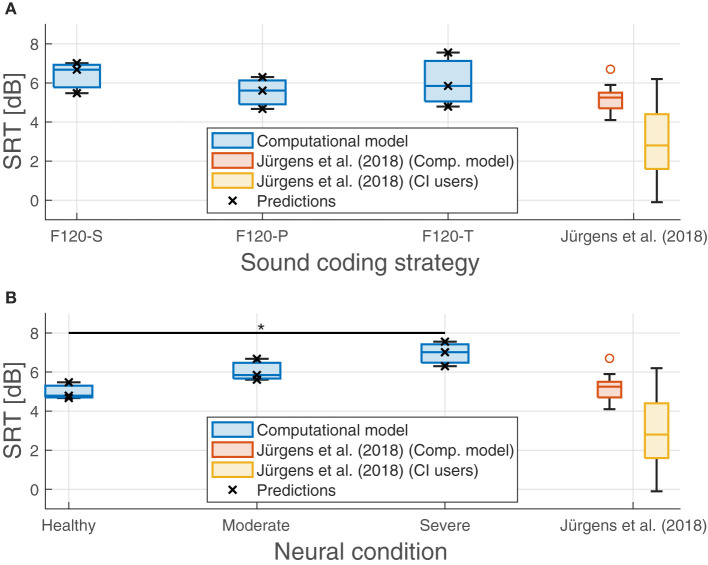
Predicted speech reception threshold (SRT) obtained in the experiments. **(A)** Performance of the proposed computational grouped by sound coding strategy. **(B)** Performance of the computational model grouped by neural health conditions. Predicted values are shown with crosses. Results from Jürgens et al. (2018) are shown as a reference in both panels as a reference. The red boxes show their predicted SRTs using an individualized model using the electrical field spread of real CI users and the yellow boxes show their measured SRTs. The symbol “*” denotes a statistical significance with a *p*-value less than 0.05 between healthy condition and severe degeneration.

The effect of neural health is visible in the overall performance, affecting not only the SRT, but also the predicted performance in quiet. Figure 11 shows the SRT grouped by sound coding strategy (Figure 11A) and neural health condition (Figure 11B). In general, worse neural health conditions led to poorer performance (higher SRTs). The differences in SRT between healthy and severe degeneration conditions for F120-S, F120-P, and F120-T were 1.53, 1.63, and 2.77 dB_SNR_, respectively. This indicates that simultaneous stimulation with F120-T was more sensitive to the effects of neural health degeneration than the F120-P.

On the contrary, the sound coding strategy that stimulated with two virtual channels simultaneously (F120-P) showed better overall performance, as shown in Figure 11, part A. With better neural health conditions, F120-T obtained better performance than F120-S, but with severe degeneration, the performance with F120-T felt below F120-S performance.

In addition, Figure 11 shows the results obtained by Jürgens et al. (2018). They measured the SRT in a group of 14 CI users using the advanced combinational encoder (ACE) sound coding strategy, which does not use current steering but it is comparable to F120-S because it uses sequential stimulation of biphasic pulses. In addition, they used a computational model to predict the performance of the CI users based on their individualized electrical field spread. Their measured SRTs ranged between −0.1 and 6.2 dB_SNR_. In contrast, the performance of their computational model ranged between 4.67 and 7.56 dB_SNR_, which is considered a poor performance according to Jürgens et al. (2018).

## 4. Discussion

In this study, a novel computational model to simulate the performance of CI users in psychoacoustic experiments was proposed. The proposed model consists of two main parts: (i) a front-end that includes a peripheral auditory model; (ii) a back-end based on the framework FADE. The peripheral auditory model combined a three-dimensional representation of the electrode-nerve interface taken from Nogueira et al. (2016), with a population of “phenomenologically” modeled ANFs taken from Joshi et al. (2017). This combination allowed to overcome the geometric limitations of the phenomenological model by making the induced current dependent on the activation function, which was obtained using the “morphological” model of Ashida and Nogueira (2018). In that sense, the proposed peripheral auditory model takes advantage of the benefits of both “phenomenological” and “physiological” approaches.

This study assessed different neural health conditions by gradually removing segments of the ANFs from the periphery toward the spiral ganglion, which is an approximation to the real physiological degeneration (Nadol, 1988; Spoendlin and Schrott, 1988; Nogueira and Ashida, 2018). The activation function decreases and widens with worse neural health conditions having an impact in the MCL levels, as shown in Figure 6. Worst neural health conditions resulted in higher MCL levels, which is consistent with the findings of Langner et al. (2021).

Predictions of the proposed computational model were obtained for two psychoacoustic experiments: SRT and SMT. The results of the SRT experiment show that worse neural health conditions result in poorer speech reception performance. Although this degeneration affected more the performance with simultaneous stimulation sound coding strategies, especially with F120-T, the detriment compared to sequential stimulation (F120-S) was rather small and not as relevant, as shown by Langner et al. (2017, 2021). A more detailed discussion regarding SMT and SRT experiments is presented in the following subsections.

### 4.1. Spectral modulation threshold experiments

As shown in Figure 9, the computational model performed similarly in SMT despite having different neural health conditions or sound coding strategies. This is because IR is a highly correlated feature along its dimensions (they are not orthogonal) and they cannot convey any relative spectral information that can be used by the HMM to differentiate between spectral rippled noise and flat noise, especially when the phase θ_0_ (see Equation 9) of the spectral rippled noise is randomized.

When the phase of the spectral ripple noise is randomized, the spectral peaks and valleys are always located in different auditory filters of the IR. With every realization of spectral ripple noise, the mean value of the IR gets closer to the mean value of the flat noise in every auditory filter. Only the standard deviation of the IR is always greater in spectral ripple noise than in flat noise. Therefore, whenever the neural activity in any auditory filter was greater, or lower, than the activity expected from the flat noise, the HMM classified it as spectral rippled noise. This may also explain why it consistently predicts an SMT around 9 dB since the loudness roving used to generate the training and testing corpus had a dynamic range of 10 dB.

Prediction algorithms based on HMMs work better with decorrelated features such as Mel frequency cepstral coefficients (MFCCs), Garbor filter bank (GFBs), or separable Garbor filter bank (SGFB) that are obtained from the spectral representation of the signal as shown by Kollmeier et al. (2016) and Schädler et al. (2016). On the contrary, IR is somehow equivalent to the bare spectrum of the audio signal, and it is highly correlation to work with HMMs.

### 4.2. Speech reception threshold experiments

Speech understanding relies on two principal aspects: temporal cues and spectral cues (Xu et al., 2005). Depending on the frequency, the temporal cues can be classified into envelopes (2–50 Hz), periodicity (50–500 Hz), and fine structure (500–10,000 Hz), the envelopes being specially important for speech understanding (Rosen, 1992). In the proposed computational model, the HMM was able to capture these envelopes along the auditory filters using Markov chains of eight states (Schädler et al., 2016); however, periodicity and fine structure is lost. The spectral cues, commonly related to the formants produced by the pronunciation of vowels and some consonants, were not properly captured by the HMM because of the before mentioned limitations of the IR when representing spectral shapes. Thus, words with relatively low SNR resulted in an IR much more similar to a word with a flat spectral shape. This may be the reason why, compared to real CI users, the performance of the computational model was worse than expected (higher SRTs) and did not account for the expected variability of around 6 dB as measured by Jürgens et al. (2018). Figure 11 also shows that this problem can be traced back to their computational model. Jürgens et al. (2018) also used the combination of IR as a feature and FADE as the back-end with an individualized electrical field spread that accounted for differences in the electrode–nerve interface between participants. However, the overall simulated performance resulted in higher SRTs than the one measured in real CI users as in our simulations.

In addition, there is a discrepancy between the effects of channel interaction in simultaneous stimulation (F120-P and F120-T) compared to sequential stimulation (F120-S). According to Langner et al. (2017, 2021), the performance with F120-S is similar to the performance with F120-P, but better than the performance with F120-T. This is not the case in the results shown in Figure 11, part A. This may be caused by the sharp decay of the voltage spread that is inversely proportional to the distance *d*_*n*_*f*__*a*__ (see Equation 1). A sharp decay diminishes the effect of electrical interaction between virtual channels in simultaneous stimulation; therefore, the performance may be more affected by the refractory period of ANFs. Notice in Figure 1 that with F120-S the electrical stimulation is continuous, while with F120-P and F120-T, there are stimulation gaps that may help ANFs to recover from refractoriness.

However, Figure 7 shows that the presented computational model is capable of reproducing the effects of electrical interaction between virtual channels. This effect is larger with the F120-T sound coding strategy because the virtual channels are closer together. Electrical interaction obtained with F120-P is much lower compared to F120-T. This may explain why in real CI users the performance with F120-P is similar to the performance with F120-S, but considerably worse with F120-T (Langner et al., 2017, 2020b). Figures 7, 11, part B, also support the hypothesis assessed in this study since the performance with triplet stimulation was significantly affected by severe degeneration compared to healthy conditions.

### 4.3. Future improvements

#### 4.3.1. Feature extraction and back-end

As discussed earlier, the IR proposed by Fredelake and Hohmann (2012) may not be the best set of features to use with the HMM already implemented in FADE because IR is highly correlated within its own dimensions. Therefore, it does not carry any spectral shape information that indicates the relative neural activity between the auditory filters. A solution could be to incorporate other decorrelated features that have been shown to improve the performance of automatic speech recognition (ASR) algorithms and that also worked with neural activity (Holmberg et al., 2005, 2007; Nogueira et al., 2007). But, although these algorithms may provide benefits in performance for the computational model, these may not represent any particular physiological aspect of the auditory system, which was the idea behind the proposed computational model.

The central processes that occur in the auditory pathway beyond the peripheral auditory system are not completely understood. The IR is a simple model that makes many assumptions about how spikes are processed centrally to interpret sounds. However, the IR was necessary to accommodate the neural activity to the temporal resolution and number of features adequate for an HMM back-end. Another way to approach this problem would be to substitute FADE as the back-end for an algorithm that can perform predictions directly from the spike activity coming from the peripheral auditory model (Alvarez and Nogueira, 2022), but this is a challenging task due to the amount of ANFs modeled and the sample rate of the spike activity. Artificial neural networks (ANNs) seem to suit well with this approach (Kell et al., 2018; Santana et al., 2018; Wang et al., 2018) since ANNs can manage a large amount of data and there is no intrinsic assumption of any central auditory process. Neural networks could function as a “black box” while the detailed modeling is focused on the peripheral auditory system (Brochier et al., 2022).

#### 4.3.2. Peripheral auditory model

Although the proposed peripheral auditory model can account for many physiological aspects, there is room for improvement.

The voltage spread obtained in this study presents a decay inversely proportional to the distance. This results in a sharper decay compared to the exponential decay measured in laboratories with saline solutions (O'leary et al., 1985; Kral et al., 1998). The sharper the decay, the less electrical interaction between channels in simultaneous stimulation; therefore, the model is less sensitive to the differences between the F120 variants studied. Nevertheless, a more realistic voltage spread could be obtained by using finite element method (FEM) or a boundary element method (BEM) in the three-dimensional electrode–nerve instead of assuming a homogeneous medium (Kalkman et al., 2014, 2022; Nogueira et al., 2016; Croner et al., 2022). This improvement of the peripheral auditory model would take into account the electrical characteristics of the bones, tissue, and other media present in the cochlea.

Regarding the ANF model, the morphological model used in this study considers the ANF as a cable with homogeneously distributed segments that do not differentiate between peripheral axon, central axon, or the soma. This approach worked because it allowed us to adjust the induced current *I* for the physiological model of Joshi et al. (2017) depending on different aspects such as electrode and nerve position, the direction of the ANF with respect to the electrode, and the effects of degeneration. A more realistic multi-compartment cable model that represents its morphology and physiology more accurately (Rattay et al., 2013; Bachmaier et al., 2019; Kalkman et al., 2022) could be implemented; however, one must be careful when coupling it with the model of Joshi et al. (2017). If the morphology already takes into account differences between the axons and the soma, the original parameters used in Equation (5) are not validated anymore.

Another improvement could be a new degeneration method that takes into account, not only the progressive inhibition of nodes, but the diameter reduction in the axons (Heshmat et al., 2020; Croner et al., 2022). In the current degeneration method, the number of degenerated nodes is not equal across the ANF population, instead, the number of nodes degenerated in each ANF is governed by a mean value and a standard deviation that is arbitrarily set to three nodes. This is an assumption made since there is no available information on how it is in real implanted cochleas. In fact, it is probable that the fibers degenerate in different patterns, for example, more degeneration in the basal turn than in the apical turns. Therefore, in further studies related to degeneration, different patterns should be taken into account including dead regions in the cochlea, which are relevant in electrical stimulation (Moore et al., 2010).

## 5. Conclusion

The computational model presented in this study was capable of executing simulations of SRT and SMT experiments. It consisted of a peripheral auditory model with a three-dimensional electrode–nerve interface that allows to represent different neural health conditions by applying a systematic degeneration to the modeled ANFs. The neural health condition affected the fitting procedure and speech reception in the expected manner, augmenting the current needed to reach MCLs and obtaining worst (higher) SRTs, respectively.

The computational model could not reproduce quantitatively the expected results in SRT experiments from real CI users where simultaneous stimulation sound coding strategies (F120-P and F120-T) consistently performed worse than sequential stimulation sound coding strategies (F120-S). Nevertheless, the results showed that the qualitative performance detriment due to neural health conditions with simultaneous stimulation (especially with F120-T) was higher than with sequential stimulation.

SMT experiments with the computational model were inconclusive since the results showed no relevant impact neither from neural health conditions nor channel interaction caused by the simultaneous stimulation. This is arguably caused by the selected IR resulting in features that did not convey spectral shape information, together with an HMM-based recognizer.

Future developments of the computational model could offer a reliable tool to assess the effects of different sound coding strategies and different neural health conditions in psychoacoustic experiments without the need for testing in implanted volunteers, especially, in the early development stages of new CI technologies. The improvements should be focused on the physiological model of the ANFs and the feature extraction from the neural activity.

## Data availability statement

The original contributions presented in the study are included in the article/supplementary material, further inquiries can be directed to the corresponding author.

## Author contributions

FA designed the experiments, programmed and integrated the computational model, verified and analyzed the results, and wrote the original manuscript draft. DK developed the auditory nerve fiber model. WN supervised the research project. All authors contributed to conceptualization, contributed to manuscript revision, read, and approved the submitted version.
